# DNAscent v2: detecting replication forks in nanopore sequencing data with deep learning

**DOI:** 10.1186/s12864-021-07736-6

**Published:** 2021-06-09

**Authors:** Michael A. Boemo

**Affiliations:** Department of Pathology, University of Cambridge, Cambridge, UK

**Keywords:** DNA replication, Residual neural networks, Oxford nanopore, DNAscent, Replication origins, Replication forks, Budding yeast

## Abstract

**Background:**

Measuring DNA replication dynamics with high throughput and single-molecule resolution is critical for understanding both the basic biology behind how cells replicate their DNA and how DNA replication can be used as a therapeutic target for diseases like cancer. In recent years, the detection of base analogues in Oxford Nanopore Technologies (ONT) sequencing reads has become a promising new method to supersede existing single-molecule methods such as DNA fibre analysis: ONT sequencing yields long reads with high throughput, and sequenced molecules can be mapped to the genome using standard sequence alignment software.

**Results:**

This paper introduces DNAscent v2, software that uses a residual neural network to achieve fast, accurate detection of the thymidine analogue BrdU with single-nucleotide resolution. DNAscent v2 also comes equipped with an autoencoder that interprets the pattern of BrdU incorporation on each ONT-sequenced molecule into replication fork direction to call the location of replication origins termination sites. DNAscent v2 surpasses previous versions of DNAscent in BrdU calling accuracy, origin calling accuracy, speed, and versatility across different experimental protocols. Unlike NanoMod, DNAscent v2 positively identifies BrdU without the need for sequencing unmodified DNA. Unlike RepNano, DNAscent v2 calls BrdU with single-nucleotide resolution and detects more origins than RepNano from the same sequencing data. DNAscent v2 is open-source and available at https://github.com/MBoemo/DNAscent.

**Conclusions:**

This paper shows that DNAscent v2 is the new state-of-the-art in the high-throughput, single-molecule detection of replication fork dynamics. These improvements in DNAscent v2 mark an important step towards measuring DNA replication dynamics in large genomes with single-molecule resolution. Looking forward, the increase in accuracy in single-nucleotide resolution BrdU calls will also allow DNAscent v2 to branch out into other areas of genome stability research, particularly the detection of DNA repair.

**Supplementary Information:**

The online version contains supplementary material available at (10.1186/s12864-021-07736-6).

## Background

Regions of a eukaryote’s genome may tend to replicate early or late in S-phase on average, but there is significant cell-to-cell heterogeneity that stems from both the set of origins used and time at which they fire [1]. The high-throughput detection of replication fork movement with single-molecule resolution is critical for understanding how a cell replicates its DNA, which is particularly important for diseases like cancer where DNA replication is a therapeutic target [2]. Oxford Nanopore Technologies (ONT) sequencing has emerged as a cost-effective platform for the detection of DNA base modifications such as 5-methylcytosine on long single molecules [3–7]. We and others have shown that halogenated bases are also detectable in ONT sequencing data [8–11]. When these bases are pulsed into S-phase cells, they are incorporated into nascent DNA by replication forks. Sequencing with ONT and detecting the position of these bases reveals a footprint of replication fork movement on each sequenced molecule, allowing this method to answer questions that would have been traditionally addressed with DNA fibre analysis but with higher-throughput and the ability to map each sequenced read to the genome. DNAscent (v1 and earlier) uses a hidden Markov model to assign a likelihood of BrdU to each thymidine [9], RepNano uses a convolutional neural network to estimate the fraction of thymidines substituted for BrdU in rolling 96-bp windows [10], and NanoMod compares modified and unmodified DNA to detect base analogues [7, 8].

This paper introduces DNAscent v2 which uses a new residual neural network architecture to assign a probability of BrdU to each thymidine. Overhauling the BrdU detection algorithm from a hidden Markov model to a residual neural network results in high-accuracy BrdU calls (95.7% balanced accuracy; 99.3% specificity; see Section S1 and Tables S1-S2 in Additional file 1) that enables the detection of replication dynamics with up to single-nucleotide resolution. DNAscent v2 supports BrdU detection on GPUs, providing the speed increase necessary to create genome-wide maps of replication dynamics in large genomes, as well as an autoencoder that automatically detects replication forks, origins, and termination sites at any point in S-phase and across different experimental protocols. This work demonstrates that DNAscent v2 is the new state-of-the-art to support DNA replication and genome stability research.

## Implementation

**Fig. 1:**
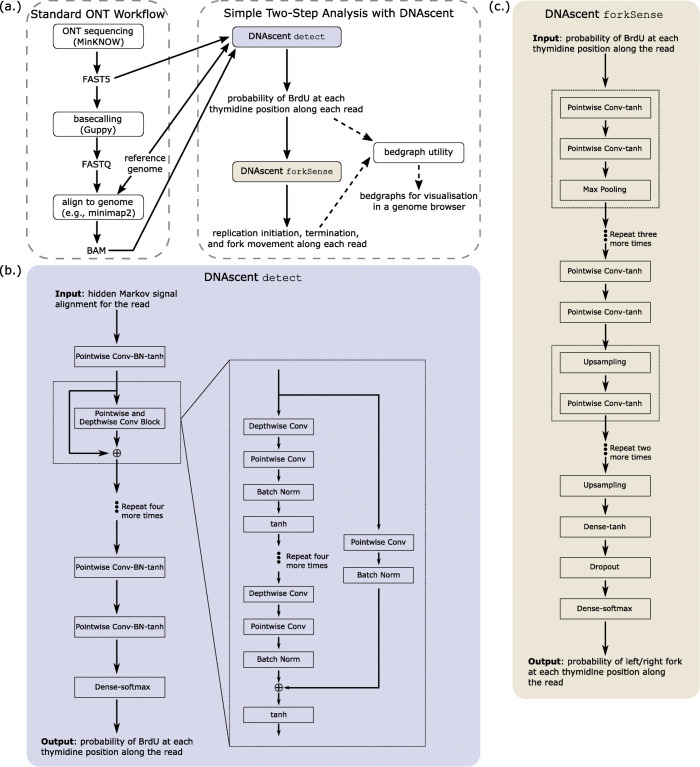
Schematic of the DNAscent v2 workflow. **(a)** A typical ONT sequencing workflow is shown, where a library is sequenced, basecalled, and aligned to a reference genome. The raw nanopore signal (in FAST5 format), the reference genome (in FASTA format), and the alignment of reads to the reference genome (in BAM format) produced during this workflow are required inputs for the DNAscent detect subprogram. DNAscent detect uses a residual neural network to assign the probability of BrdU at each thymidine position in each read. These probabilities are written to a single file which is the only input for the DNAscent forkSense subprogram. DNAscent forkSense uses an autoencoder neural network to interpret the pattern of BrdU incorporation on each read into fork direction, and replication origin, fork, and termiantion calls are written to bed files. As an optional third step, DNAscent comes equipped with a utility that can convert the output of DNAscent detect and forkSense into bedgraphs that can be visualised in a genome browser. **(b)** Architecture of the residual neural network used by DNAscent detect, loosely based on [16]. For each read, DNAscent detect performs a hidden Markov signal alignment to create an input tensor for the neural network. The final softmax layer normalises the output of the network to the probability that BrdU is at each thymidine position in the read. Further details, training information, and the number of parameters used in each layer are described in Section S2 of Additional file 1. **(c)** Architecture of the autoencoder neural network used by DNAscent forkSense. For each read, the output of DNAscent detect (the probability of BrdU at each thymidine position along the read) forms the input tensor, and the network outputs the probability that a leftward- and rightward-moving fork passed through each thymidine position on the read during the BrdU pulse. Further details, training information, and the number of parameters used in each layer are described in Section S3 of Additional file 1. Abbreviations: batch normalisation (BN), convolution (Conv)

The DNAscent v2 software consists of a simple two-step analysis pipeline requiring only three easy-to-make inputs: the FAST5 files containing raw signal data (produced by ONT’s MinKNOW software during sequencing), a reference genome, and the alignment (in BAM format) of ONT reads to the genome (Fig. 1a). The subprogram detect in DNAscent v2 uses these inputs to call the probability of BrdU at each thymidine position for each sequenced molecule. These probabilities are written to a single output file in a table format that was designed to be easy to parse. The output file from DNAscent detect is the only input for a new subprogram called forkSense that interprets the pattern of BrdU incorporation on each read to determine the probabilities that a leftward- and rightward-moving fork passed through each position during the BrdU pulse.

**Fig. 2:**
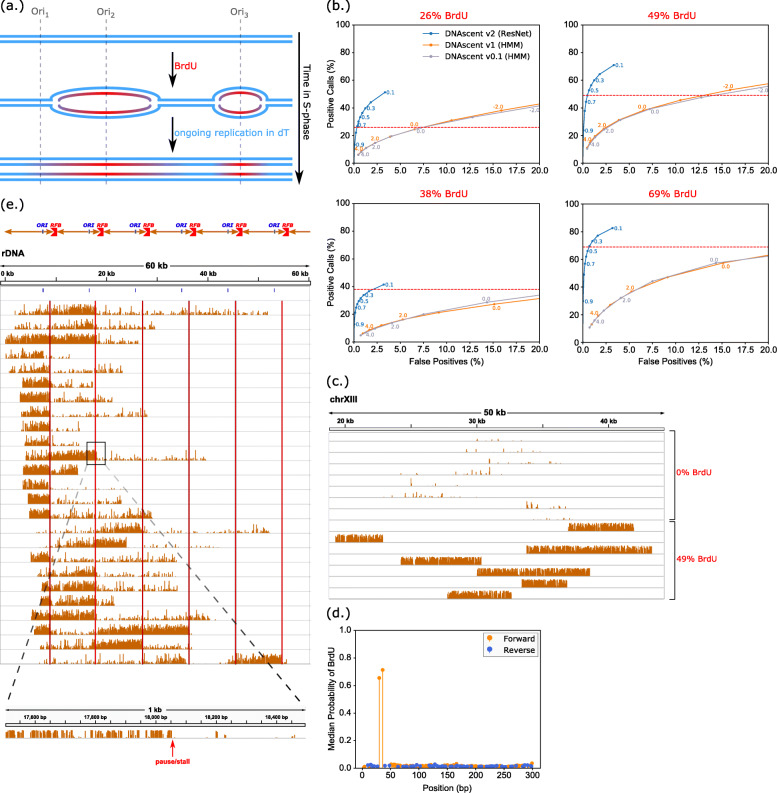
Performance of the DNAscent v2 detect subprogram. **(a)** When the thymidine analogue BrdU is pulsed into S-phase cells, BrdU is incorporated into the newly replicated nascent DNA in place of thymidine. Detecting BrdU in nascent DNA sequenced with ONT can reveal the movement of replication forks in millions of single molecules. **(b)** ROC curves showing the ratio of positive BrdU calls to false positive BrdU calls for four different experiments with different BrdU-for-thymidine substitution rates. The 26% and 49% BrdU samples are from [9] while the 38% and 69% samples are from [10]. The BrdU-for-thymidine substitution rate as measured by mass spectrometry is indicated by the dashed red line. Points along each curve are different thresholds above which a BrdU call is considered positive; for DNAscent v2, these are probabilities whereas for DNAscent v1 and below, these are log-likelihoods. Each curve was calculated using 5,000 reads. The x-axis of each plot has been truncated from 0-100% to 0-20% for clarity. Only results for DNAscent are shown, as RepNano does not call BrdU with single-nucleotide resolution. **(c)** Bedgraphs visualised in IGV [12] showing the proability of BrdU called at each thymidine position for a randomly selected subset of reads used in the 49% BrdU ROC curve analysis. Each track is a single read, and the y-axis of each track ranges from 0 to 1. **(d)** The median probability of BrdU called by DNAscent v2 at each thymidine position along primer extension reads (N=273) from [9] where BrdU has been substituted for thymidine in two known positions (30 and 36 bp) on the forward strand. All other positions on the forward and reverse strand are unsubstituted. **(e)***S. cerevisiae* rDNA consists of 150-200 9.3 kb repeats, each of which has an origin of replication (top track) and a replication fork barrier (vertical lines) that block rightward-moving forks. There is a sharp drop-off in BrdU incorporation where rightward-moving forks hit the barrier, indicating a fork pause or stall. A total of 25 reads are shown, and for a representative read, the inset zooms in on a 1 kb region that includes the barrier

The subprogram detect in DNAscent v2 detects BrdU with single-nucleotide resolution using a residual neural network consisting of depthwise and pointwise convolutions (Fig. 1b; see Section S2, Figure S1, and Table S3 in Additional file 1 for details). The model was trained using nanopore-sequenced genomic DNA from a *S. cerevisiae* thymidine auxotroph [9]. In particular, the training material consisted of unsubstituted DNA as well DNA with 80% BrdU-for-thymidine substitution (Figure S2 in Additional file 1). A shortcoming of earlier DNAscent versions was that origin calling was designed to work in synchronised early S-phase cells. To that end, DNAscent v2 includes a new subprogram called forkSense that was designed to work in both synchronous and asynchronous cells at any point in S-phase. forkSense uses an autoencoder neural network to assign the probabilities that a leftward- and rightward-moving fork passed through each position on a read during the BrdU pulse (Fig. 1c; see Section S3, Figures S3-S4, and Table S4 in Additional file 1 for details). forkSense matches up converging and diverging forks in order to call confidence intervals of replication origins and termination sites on each nanopore-sequenced molecule. Hence, DNAscent detect and forkSense together are able to identify the BrdU “footprint” of replication forks on each nanopore-sequenced molecule (Fig. 2a).

**Fig. 3:**
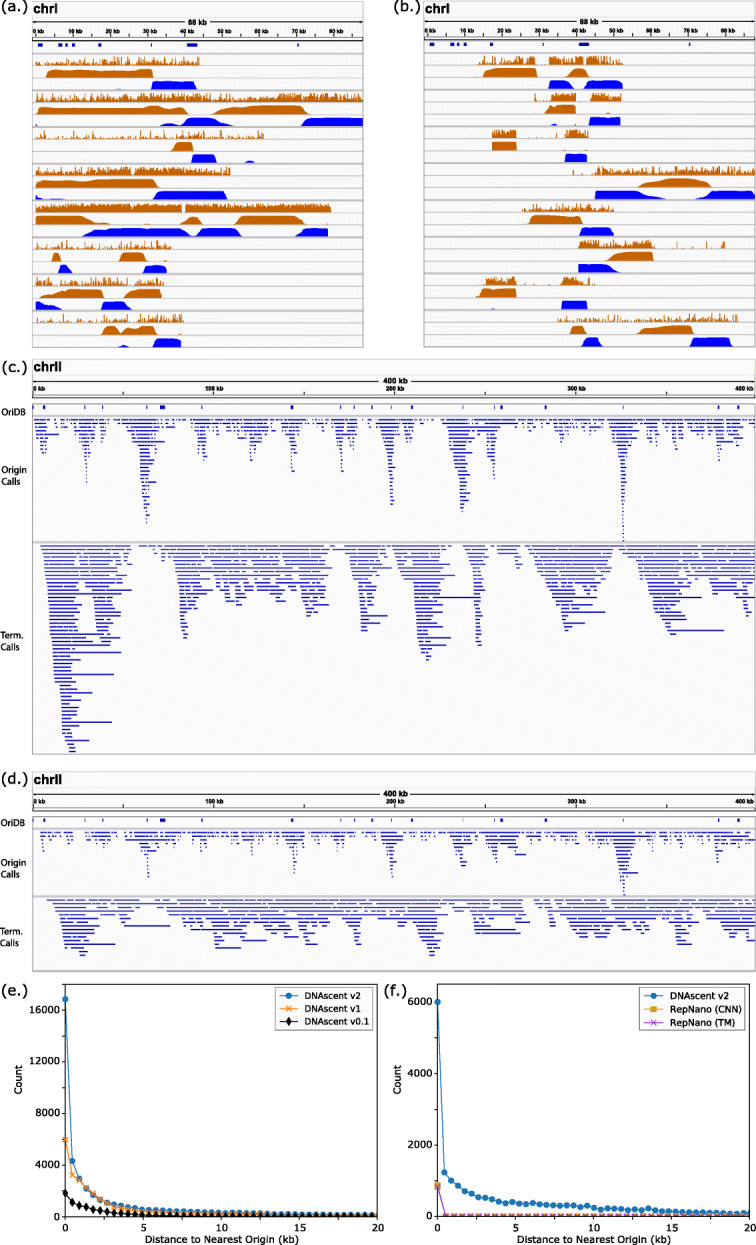
Performance of the DNAscent v2 forkSense subprogram. **(a-b)** Individual reads mapping to *S. cerevisiae* chromosome I are shown for *S. cerevisiae* cells **(a)** synchronised in G1 and released into BrdU and **(b)** asynchronous and pulsed with BrdU and chased with thymidine. Origins that are confirmed and likely from OriDB are shown in the top track. Eight reads are shown for each experiment where each read is represented as a group of three tracks: the probability of BrdU at each thymidine (upper track; from DNAscent detect) and the probability that a leftward-moving fork (middle track; from DNAscent forkSense) and rightward-moving fork (lower track; from DNAscent forkSense) passed through each position during the BrdU pulse. The y-axis of each track ranges from 0 to 1. **(c-d)** Pileup of all replication origins and termination sites called by forkSense that mapped to *S. cerevisiae* chromosome II for *S. cerevisiae* cells **(c)** synchronised in G1 and released into BrdU (2,980 origin calls from a total of 9,864 reads) and **(d)** asynchronous and pulsed with BrdU and chased with thymidine (1,461 origin calls from a total of 5,186 reads). Only reads with mapping length ≥20 kb and mapping quality ≥20 were used. The OriDB track shows confirmed and likely origins. For clarity, only the first 400 kb of chromosome II are shown; the full length of chromosome II is shown in Figure S5 in Additional file 1. **(e)** Distribution of the distance between each origin call and the nearest confirmed or likely origin from OriDB for *S. cerevisiae* cells synchronised in G1 and released into BrdU. The results of three versions of DNAscent are shown. RepNano only made a total of 14 origin calls on this dataset when run with the default settings, so these were omitted for clarity. **(f)** A similar analysis to Fig. 3e, but for asynchronous *S. cerevisiae* cells pulsed with BrdU and chased with thymidine. Results for DNAscent v2 are shown alongside results from the RepNano transition matrix (TM) and convolutional neural network (CNN) origin calling algorithms run using the default parameters. Earlier versions of DNAscent were not designed to call origins in asynchronous cells, so only the results from DNAscent v2 are shown

In addition to improving performance and adding functionality, DNAscent v2 development placed a particular focus on ease-of-use and accessibility for laboratories that may not have access to computational scientists or bioinformaticians. Origin calling with RepNano has fourteen adjustable parameters and earlier versions of DNAscent have three, but forkSense in DNAscent v2 does not require any tuning. DNAscent v2 also comes packaged with a utility that converts the outputs of detect and forkSense into bedgraphs such that BrdU and fork probabilities can easily be viewed side-by-side for each read (as in Fig. 3a-b) in the Integrative Genomics Viewer (IGV) [12] or the UCSC Genome Browser (http://genome.ucsc.edu) [13], and origin, termination, and fork calls are likewise written to bed files. To support the genome-wide measurement of replication dynamics in organisms with larger genomes, DNAscent v2 can optionally run BrdU detection on a GPU and benchmarks approximately 4.5 × faster than DNAscent v1 and approximately 3.5 × faster than RepNano (see Section S4 and Tables S5-S7 in Additional file 1).

## Results

To evaluate the performance of DNAscent detect, receiver operator characteristic (ROC) curves were plotted using nanopore sequenced unsubstituted DNA to measure false positives and DNA with four different BrdU-for-thymidine substitution rates (Fig. 2b). DNAscent v2 outperformed the previous versions of DNAscent by a wide margin in all four samples. Bedgraphs of the probability of BrdU at each thymidine position for a subset of unsubstituted reads and 49% BrdU-for-thymidine substituted reads from the ROC curve analysis are shown in Fig. 2c, highlighting the difference between substituted and unsustituted reads. In concordance with the ROC curves, unsubstituted reads are largely devoid of false positives. To show that DNAscent v2 distinguishes BrdU from thymidine with single-nucleotide resolution, BrdU detection was run on substrates with two BrdU bases at known positions [9] where DNAscent v2 was able to clearly identify the positions of both BrdU bases (Fig. 2d). This accurate single-nucleotide resolution is particularly important for genome stability applications such as identifying the precise location of replication fork stalls; we previously detected fork pausing/stalling at replication fork barriers in *S. cerevisiae* rDNA with 2-kilobase (kb) resolution using DNAscent v0.1 [9], but DNAscent v2 can detect sites of fork pausing/stalling with single-nucleotide resolution (Fig. 2e). With DNAscent v2, the BrdU calls are clean enough that the single-nucleotide resolution BrdU calls can be visualised directly as bedgraphs in IGV [12] without the need for any smoothing or further processing from the software.

DNAscent forkSense was tested on two different BrdU-pulse experimental protocols: *S. cerevisiae* cells that were synchronised in G1 and released into S-phase in the presence of BrdU with no thymidine chase [9] and asynchronous thymidine-auxotrophic *S. cerevisiae* cells where BrdU was pulsed for 4 minutes followed by a thymidine chase [10]. Example single molecules mapping to a region that includes several efficient origins on *S. cerevisiae* chromosome I are shown for both experiments (Fig. 3a-b). forkSense calls origins as the regions between diverging leftward- and rightward-moving forks and calls termination sites as the regions between converging forks. A pileup of replication origins and termination sites called on *S. cerevisiae* chromosome II is shown for cells synchronised in G1 (Fig. 3c; Figure S5c in Additional file 1) and asynchronous cells (Fig. 3d; Figure S5d in Additional file 1). While the location of called replication origins shows good agreement with confirmed and likely origins from OriDB [14] in both cases (Fig. 3e-f) this work corroborates the findings of [9, 10] that high-throughput, single-molecule analysis reveals replication origins that are far (>5 kb) away from previously annotated origins. DNAscent v2 is able to capitalise on its improved BrdU detection to detect several fold more origins than both previous versions of DNAscent and RepNano (Fig. 3e-f).

## Discussion

While several tools have been developed in recent years that can detect BrdU in Oxford Nanopore reads, DNAscent v2 has a number of key advantages. Unlike NanoMod [7], DNAscent v2 is able to positively identify BrdU without the need for sequencing both BrdU-substituted and unsubstituted DNA that covers the same region of the genome. Unlike RepNano [10], DNAscent v2 can call BrdU with single-nucleotide resolution which is critical for accurately detecting sites of fork stalling and the genomic features (e.g., DNA sequence motifs or replication-transcription collisions) that may have caused aberrant fork movement. Importantly, DNAscent v2 far surpasses its previous major releases (v1 and earlier) [9] in accuracy of BrdU calling (Fig. 2b), resolution of detecting sites of fork pausing/stalling (Fig. 2e), accuracy of origin calling (Fig. 3e), and its ability to now detect replication forks at any point in S-phase (Fig. 3b,f). The improvement to single-nucleotide resolution BrdU calling in detect, together with the forkSense algorithm, has allowed DNAscent v2 to make significantly more origin calls than previous versions when run on the same data set, and as shown by Fig. 3e, most of these additional calls were near confirmed and likely origin sites. This suggests a decrease in false negative origin calls, enabling DNAscent v2 to create a more accurate picture of how replication took place on each individual molecule. When analysing all nanopore-sequenced molecules together, these improvements mean that less data is required to create whole-genome maps of replication origin and termination site locations, which is particularly important for studying replication in larger genomes.

Transitioning the DNAscent detect BrdU calling algorithm from the hidden Markov forward algorithm to a new residual neural network architecture has increased the accuracy of single-nucleotide resolution BrdU calling, making this new version of DNAscent applicable to more areas of genome stability research. The accuracy shown in Fig. 2 indicates that DNAscent v2 should be able to detect sites of DNA repair, where accurate BrdU calls within very short (1-10 inserted nucleotides for base excision repair and about 30 nucleotides for nucleotide excision repair) would be critical. The residual neural network in DNAscent v2 also creates a more natural platform for future work on the detection of multiple base analogues and/or base modifications in the same molecule. DNA fibre analysis relies on sequential pulses of different base analogues to determine fork direction while DNAscent currently determines fork direction from the changing frequency of BrdU-for-thymidine substitution across a molecule. While DNAscent’s current single-analogue approach is advantageous in its simplicity, the detection of multiple analogues would be necessary to answer certain questions typically addressed with fibre analysis, such as the stability of stalled replication forks [15].

## Conclusions

This paper has introduced DNAscent v2, which utilises residual neural networks to significantly improve the single-nucleotide accuracy of BrdU calling compared with the hidden Markov approach utilised in earlier versions. DNAscent v2 also includes the new forkSense subprogram which uses an autoencoder to infer the movement of replication forks from patterns of BrdU incorporation. forkSense can call the location of replication forks, origins, and termination sites in single-molecules across a range of experimental protocols with a sensitivity that exceeds both earlier versions and other competing tools. These new methodologies, together with improvements in speed and ease-of-use, make this technology an important new piece of the toolkit in DNA replication and genome stability research.

## Availability and requirements

**Project name:** DNAscent**Project home page:**https://github.com/MBoemo/DNAscent**Operating system(s):** Linux**Programming language:** C, C++, Python**Other requirements:** GCC 6.1 or higher, CUDA 10.0 and cuDNN 7.5 (for GPU use only)**License:** GNU GPL-3.0**Any restrictions to use by non-academics:** None

## Supplementary Information

**Additional file 1** Supplementary information. The supplementary information provides technical details about how the neural networks in DNAscent v2 were designed and trained. Details are also provided for the runtime comparisons mentioned in the text.

## Data Availability

DNAscent v2 is open-source under GPL-3.0 and is available at https://github.com/MBoemo/DNAscent. ONT sequencing data for BrdU detection training, primer extension, and synchronised cell cycle experiments were released with [9] in NCBI GEO under accession number GSE121941. ONT sequencing data for the asynchronous cell cycle experiment was released with [10] in ENA under accession number PRJEB36782 (experiment ERX4016778).

## Abbreviations

ONT: Oxford nanopore technologies
ROC: Receiver operator characteristic
BN: Batch normalisation
CONV: Convolution
TM: Transition matrix
CNN: Convolutional neural network
IGV: Integrative Genomics Viewer
